# Epoxy-Based Blend Formulation for Dual Curing in Liquid Crystal Display 3D Printing: A Study on Thermomechanical Properties Variation for Enhanced Printability

**DOI:** 10.3390/polym16030358

**Published:** 2024-01-29

**Authors:** Claudio Tosto, Lorena Saitta, Alberta Latteri, Gianluca Cicala

**Affiliations:** Department of Civil Engineering and Architecture, University of Catania, Viale Andrea Doria 6, 95125 Catania, Italy; lorena.saitta@phd.unict.it (L.S.); alberta.latteri@unict.it (A.L.); gianluca.cicala@unict.it (G.C.)

**Keywords:** epoxy resin blends, photocurable acrylate, printability, thermal curing performance, LCD printing, additive manufacturing

## Abstract

The aim of this study was to explore the thermal properties of epoxy–acrylate blends for the liquid crystal display (LCD) 3D printing technique. Starting from an epoxy–acrylate blend with a ratio of epoxy to acrylate of 50:50, the effect of adding a reactive monofunctional epoxy diluent was evaluated. The diluent was a resin composed by oxirane, mono[(C12-14 alkyl) methyl] derivatives selected for its low viscosity (i.e., 1.8 Poise) at room temperature and its reactivity. The diluent content varied from 15 to 25 wt% and, for all the formulation, double curing cycles, where thermal curing followed photocuring, were studied. The effect of different curing temperatures was also evaluated. The control of the diluent content and of the curing temperature allowed tailoring of the thermomechanical resin properties while improving the resin’s processability. The glass transition ranged from 115.4 °C to 90.8 °C depending on the combination of diluent content and post-curing temperature. The resin developed displayed a faster processing time tested on a reference part with printing time of 4 h and 20 min that was much lower than the printing times (7 and 16 h) observed for the starting formulations.

## 1. Introduction

Three-dimensional printing has revolutionized the manufacturing landscape, offering a plethora of techniques with diverse physical principles and material compositions. Among these cutting-edge technologies, Liquid Crystal Display (LCD) printing has garnered considerable attention due to its simplicity, cost effectiveness, and ability to achieve high-resolution prints [1,2,3]. As a result, it has found widespread applications in diverse fields, including dentistry, microfluidic, jewelry design, toy manufacturing, and even composite tooling [4,5,6,7,8,9]. The core principle behind LCD printing involves the use of photosensitive resins that solidify when exposed to light, daylight, or UV light [10]. Of the various photosensitive resins utilized, acrylate and methacrylate-based materials are widely used, as their photopolymerization relies on free-radical initiation and catalyzed reactions [11,12].

To address some of the challenges associated with photopolymerization, epoxy resins have been incorporated as co-reactants in the formulation [13,14,15,16]. Initially introduced to control shrinkage during curing, researchers have now begun to explore the potential of epoxy resins in enhancing the thermomechanical performance of LCD-printed objects through dual-curing strategies [13,16,17,18]. Furthermore, novel formulations have been developed to harness the synergistic benefits of both acrylate and epoxy chemistries, aiming to create materials with superior mechanical properties and broader application potential [11,16]. In a recent review Fernàndez-Francos et al. [19] discussed the potential of dual curing as an alternative approach to enhance the properties of cured parts in single- or multi-stage processing scenarios. Notably, results were achieved using this approach as presented in recent research. For example, Binyamin et al. [20] synthetized Bisphenol A epoxidemonoacrylate starting from diglycidyl ether bisphenol A (DGEBA) that was dual cured, obtaining a system showing a glass transition temperature (*T*_g_) of 241 °C. In the same paper, adding a sol–gel precursor containing photocurable groups enabled the in situ formation of SiO_2_ particles, resulting in a cured resin displaying a remarkable *T*_g_ value of 283 °C. Wang et al. [21] developed photopolymer resins mixing epoxy resin and bio-based methacrylate showing, after the double curing, an interpenetrated polymer network (IPN) structure with improved properties. Similarly, Chen et al. [22] developed a dual curing dimethacrylate/epoxy resin that, after being 3D printed, was thermally cured, showing a *T*_g_ up to 140 °C. This system used an epoxy system based on DGEBA and an ammine. In this paper, post-printing curing conditions impacted on the thermomechanical properties and the morphologies of the systems.

The use of double-curing systems is also employed commercially, as demonstrated in a remarkable example by the resin systems produced by Carbon3D systems as demonstrated, for example, in the work by Obst et al. [23].

In light of recent advancements, this paper aims to expand upon the outcomes of our prior study [16], which concentrated on the development of an epoxy–acrylate dual-curable blend specifically tailored for LCD printing. The existing blend was a result of a 50:50 mixture of a commercial acrylate system, denoted as Cream resin (*C*), and a DGEBA resin cured with diethyl-toluene diamine (DETDA). Although this blend exhibited a high *T*_g_ of 174 °C, the printing parameters (e.g., Z lift and retract speeds, Top time, etc.) resulted in prolonged printing durations (e.g., 16 h for a part with a volume of 78 cm^3^).

Innovatively building upon our previous research, this work introduces a mono-functional epoxy resin based on oxirane, mono[(C12-14 alkyl) methyl] derivatives, as an additional component to the formulation. This epoxy resin serves as a reactive diluent within the blend. The selection of this resin is driven by its low viscosity (1.8 Poise at room temperature) and its reactive nature. The inclusion of a low-viscosity component aims to reduce the overall formulation viscosity, thereby enhancing resin flow during printing and enabling faster production speeds. The choice of a reactive resin is motivated by the necessity to mitigate adverse effects on the final thermomechanical properties. The content of the reactive diluent ranged from 15 to 25 wt% in the resin system, resulting in the formation of an interpenetrating network (IPN) upon curing, owing to the reactive nature of both epoxy and acrylate systems employed.

Thanks to the inclusion of the component acting as a reactive diluent, the innovativeness of this work lies in creating printable blends via LCD printing in drastically reduced times compared to the previously examined blends, without compromising thermomechanical properties. Moreover, our study demonstrates a notable decrease in time required for the thermal curing step in the dual-curing approach.

Thermal analyses, utilizing Differential Scanning Calorimetry (DSC) and Dynamic Mechanical Analysis (DMA), were conducted to explore the impact of thermal post-curing cycles on the *T*_g_. A factorial design was implemented through the Design of Experiment (DoE) technique for this purpose. Furthermore, a speed cure test was performed on all blends to evaluate the printing conditions. Specifically, one formulation, modified with 15 wt% of the reactive diluent, underwent LCD 3D printing, and the associated process times were assessed in comparison to the previously examined blend.

## 2. Materials and Methods

### 2.1. Materials

Following the approach of our previous study [16], we employed two distinct resin systems in this research. The first utilized resin system was the commercial daylight resin, Cream Hard (referred to as *C*), which was sourced from Photocentric Ltd. (Peterborough, UK). The Cream Hard resin is an acrylate-based photosensitive material that solidifies upon exposure to natural daylight. This resin is purposely designed for the liquid crystal display (LCD) 3D printing technique.

For the second resin system, we formulated a thermally curable epoxy resin blend. This blend consisted of diglycidyl ether of bisphenol A (DGEBA) obtained from Huntsman (Basel, Switzerland) and diethyltoluene diamine (DETDA) sourced from Lonza (Basel, Switzerland). DGEBA is widely recognized for its exceptional properties and versatility in various industrial applications. When combined with DETDA, it forms a reliable and efficient curing system for epoxy resins, ensuring the formation of robust and durable printed objects. The DGEBA/DETDA system was selected because it is liquid and stable at room temperature with pot life higher than 600 min at room temperature. This epoxy system will be identified as *E* from now on.

To further tailor the properties of the epoxy–acrylate blends, we introduced a third component (identified as *B* henceforth), which is based on oxirane, mono[(C12-14 alkyl) methyl] derivatives and is characterized by a low viscosity of 1.8 Poise at room temperature. This specific component was selected for assuming the diluent role and properly tailoring the viscosity of the here-proposed blend, because its use led to excellent results in the infusion process for composite manufacturing [24].

### 2.2. Epoxy Blend Formulation

DGEBA and DETDA were combined at room temperature in a precise 2:1 stoichiometric ratio. The epoxy-based blends were formulated by combining the uncured epoxy formulation (*E*) with cream resin (*C*) in a 50:50 weight ratio at room temperature. This blending process was conducted by mechanical mixing with a centrifugal planetary mixer (ARV-310 by Thinky, Laguna Hills, CA, USA) operating at a speed of 2000 rpm, under a vacuum pressure of 0.3 kPa, and with a mixing time of 5 min. This mixing procedure resulted in the formation of a void-less and homogeneous mixture, denoted as CE5050 in accordance to our previous paper. Afterward, oxirane, mono[(C12-14 alkyl) methyl]-based resin (*B*) was incorporated at varying weight ratios, as detailed in Table 1, repeating the mechanical mixing procedure outlined above.

### 2.3. Resin Photo- and Thermal Curing

Photo curing was carried out using the LC-Precision Ceramic 1.5 LCD printer manufactured by Photocentric (Peterborough, UK) in accordance with an approach described in our previous paper [16]. This printer utilizes a 7-inch LCD screen as a display for the resin’s photocuring irradiating daylight through a transparent plastic film acting as the bottom of the vat during the printing process. The LC-Precision Ceramic was used for the speed cure test and for the preparation of the samples used for DMA analysis. The initial printing conditions for the cream resin were the same suggested by the resin manufacturers Photocentric.

The modified blends were characterized by the speed cure test to ascertain their best processing parameters. This test allowed determination of two key parameters: exposure times [s] and width overcure [%]. These parameters are an important part of our experimental procedure and provide valuable insights into the performance of the resins during the printing process. The experimental procedure for the speed cure test was detailed in a separate publication [16], which we referenced for consistency and to ensure accurate and replicable results. This test method involves subjecting the resin blends to controlled photocuring conditions, typically under specific light exposure, to observe how they respond and solidify.

Exposure times refer to the duration of light exposure required to achieve adequate curing of the resins. Understanding these exposure times is critical as they directly influence the efficiency and speed of the 3D printing process. By determining the optimal exposure times for each epoxy–acrylate blend, we aim to enhance the overall printing efficiency and reduce production time. Width overcure, on the other hand, provides essential information about the dimensional accuracy of the 3D printed objects. It refers to the extent to which the printed object’s dimensions exceed the intended design specifications. Monitoring and minimizing width overcure are crucial for achieving precise and accurate prints, especially in applications where dimensional accuracy is crucial.

As a preliminary evaluation of printability, the two parameters derived from the speed cure tests served as valuable indicators about how well the epoxy–acrylate blends performed in the LCD 3D printing process. By understanding how these resins responded to light exposure and assessing their dimensional accuracy, we identified the most suitable formulations that exhibit excellent printability and overall performance.

In this study, two thermal curing cycles were investigated. Prior to thermal curing, all formulations underwent an initial photocuring process using a silicon mask in direct contact with the LCD screen of the 3D printer. After the photocuring step, the samples were left to post-cure under natural daylight conditions for a duration of 1 day.

Subsequently, the post-cured samples were subjected to thermal curing in a standard ventilated oven, employing two distinct cure cycles:

- Isothermal cure at 140 °C for 2 h, followed by a ramp at 2 °C/min to 180 °C, and a hold at this final temperature for 2 h (referred to as ID cycle PC180).
- An alternative cure profile involving only an isothermal cure at 220 °C for a duration of 3 h (referred to as ID cycle PC220).

The data obtained from the thermal curing analyses, combined with the results from the speed cure tests, enabled a holistic evaluation of the epoxy–acrylate blends and their suitability for diverse 3D printing applications.

### 2.4. Thermomechanical Characterization

Calorimetric measurements were conducted using a Shimadzu DSC-60 instrument from Shimadzu (Kyoto, Japan). For each measurement, samples weighing between 5 and 7 mg of uncured resin were carefully placed in a 40 μL sealed aluminum crucible. The DSC analyses were performed by subjecting the samples to controlled heating, starting from room temperature, and ramping up to 350 °C at a rate of 20 °C/min. The DSC instrument recorded the exothermic heat released during the process, and the total exothermic heat was calculated based on the area under the exothermic peak.

For DMA analysis, cured specimens with dimensions of (10 × 8 × 2) mm^3^ were used. The DMA experiments were carried out using a Tritech DMA machine from Triton Ltd. (Wrexham, UK). The aim was to measure the storage modulus (E’) and the tanδ of the cured specimens. The tests were performed in single-cantilever mode with a 20 μm amplitude and a frequency of 1 Hz. To determine the *T*_g_ of the resin, DMA was conducted with a ramp test at a rate of 2 °C/min, ranging from 25 to 250 °C

To rationalize and analyze all the experimental data, a Design of Experiment (DoE) approach was performed. A factorial design involving two factors, namely, thermal curing profile and *B* component, was utilized in the study. The thermal curing factor was investigated at two levels (a=2): PC180 and PC220, which correspond to the two thermal curing cycles described in Section 2.3. The B component factor was examined at three levels (b=3): 15 wt%, 20 wt%, and 25 wt%. By systematically varying these factors and studying their interactions, we aimed to gain a deeper understanding of their influence on the material properties and performance of the epoxy–acrylate blends.

The DoE approach allows for a comprehensive and efficient exploration of the effects of multiple variables on the material characteristics. By using this method, we can optimize the resin formulations and identify the most influential factors that lead to desirable properties for 3D printing applications. The combination of DSC and DMA analyses, along with the DoE approach, strengthens our research, providing valuable insights into the thermal behavior and mechanical performance of the epoxy–acrylate blends, ultimately contributing to the advancement of additive manufacturing technologies.

### 2.5. Viscosity Estimation

The resin viscosity $\left(\eta^{*}\right)$ value is a critical parameter for LCD 3D printing, and should be lower than 100 Poise to enable flowing when the building platform moves up and down, thus creating a layer of uncured resin which is uniform in all its thickness within the vat. To prove that the developed resin formulations do not exceed this threshold value, an estimation for their viscosity was performed by mean of the lever rule and knowing the viscosity of each component blended at room temperature (see Table 2).

## 3. Results and Discussion

### 3.1. Thermomechanical Analysis

**Figure 1:**
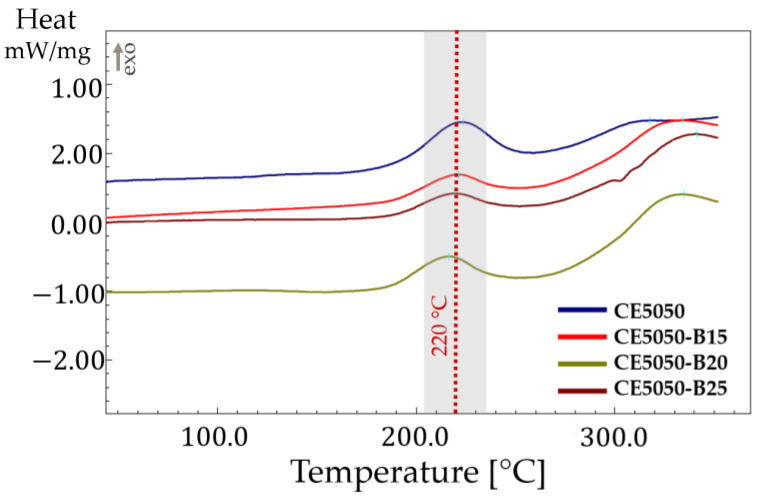
DSC thermograms of each investigated blend.

All the formulations were subjected to an in-depth analysis using DSC to elucidate their thermal behavior. A distinct exothermic peak was identified (Figure 1), prominently centered around 220 °C. This exothermic event signifies a thermally activated process within the cured resin, suggesting the occurrence of further chemical transformations at elevated temperatures.

The DSC data clearly showed that the presence of the B component did not affect the exothermic peak. Minor variations are observed in the enthalpies too. Similar results were found in the literature [24] when reactive diluents were used. The presence of the epoxy moiety on the oxirane, mono[(C12-14 alkyl) methyl]-based C resin allowed coreaction with the epoxy–amine system. However, the C resin, being mono-functional, cannot significantly impact the curing kinetic of the epoxy–amine system as was observed with di- or tri- functional epoxy diluents [25].

**Figure 2:**
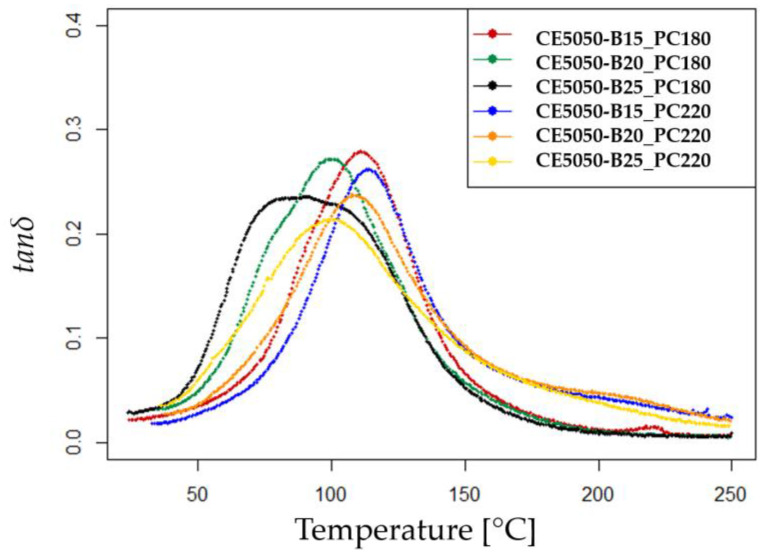
Obtained tanδ vs. temperature curves from the DMA tests.

The dynamic mechanical analysis (DMA) test results are presented in Figure 2.

The curves depict the DMA data of the post-cured samples. Notably, all the curves corresponding to the samples subjected to the PC220 thermal curing cycle show a slight shift towards higher temperature values compared to those subjected to the PC180 cycle.

The increase in the *B* component at 25 wt% leads to broader and less defined peaks, likely influencing the viscoelastic properties of the mixture and resulting in the observed DMA curve characteristics. The measured T_g_ values are reported in Table 3 and the trend with increasing *B* content is to have a decreasing T_g_. The observed behavior can be elucidated by the inherent characteristics of the *B* component. Despite its classification as a reactive diluent, the *B* component possesses merely a singular epoxy reactive group. Consequently, while the diluent is capable of engaging in the epoxy–amine reaction, it lacks the capacity to augment the cross-link density. This is in stark contrast to multifunctional epoxy diluents, which, owing to their multiple reactive groups, demonstrate an ability to enhance the *T*_g_ [24,26].

**Figure 3:**
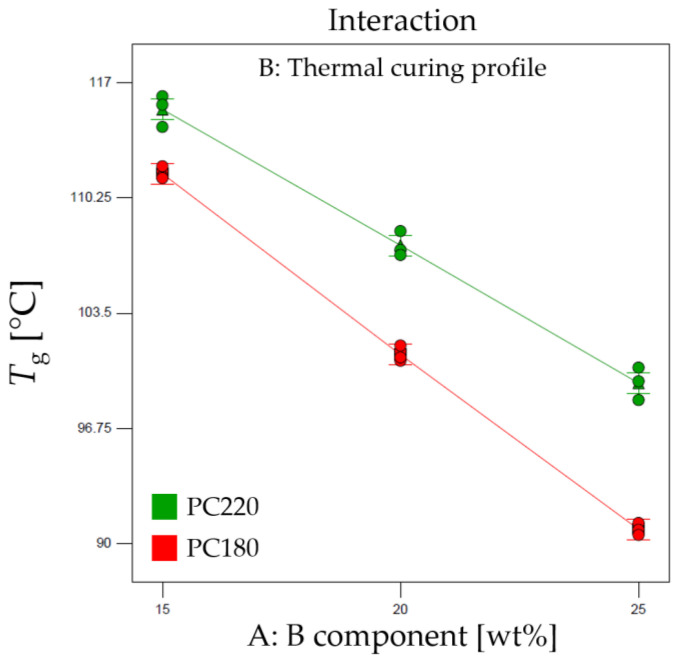
Effect diagram for the T_g_.

To gain a deeper understanding of the factors influencing the *T*_g_ observed in the DMA results, an analysis of variance (ANOVA) study was conducted. The thermal curing profile (factor A) was found to be a highly influential factor (*p*-value < 0.001). This suggests that the choice of thermal curing cycle significantly affects the T_g_ value of the epoxy–acrylate blends. Additionally, an interaction between factor A (B component) and factor B (Thermal curing profile) was observed, indicated by a significant AB interaction effect (*p*-value < 0.001) (see Figure 3).

The ANOVA study further indicates that the model employed in the analysis exhibits excellent robustness in predicting and defining the observed response, as evidenced by a high R-squared value of 0.99. The latter finding signifies that the model accurately explains the variation in the T_g_ values observed in the DMA curves.

The effect diagram reported in Figure 3 provides a visual representation of the impact of the factors on the T_g_ of the epoxy–acrylate blends. It demonstrates the influential role of the thermal curing profile (factor A) and how it interacts with the B component content (factor B) to affect the *T*_g_. By understanding these interactions, we can tailor the formulation and optimize the curing conditions to achieve the desired thermal properties in the 3D printed objects.

Comprehensive analysis of the DMA data and the ANOVA study helps elucidate the relationship between the curing profiles and the *T*_g_, providing essential insights for fine-tuning the resin formulations. These findings contribute significantly to enhancing the thermal performance and mechanical behavior of the epoxy–acrylate blends, ultimately leading to the development of high-quality and reliable 3D printed objects.

### 3.2. Viscosity (η^*^)

**Figure 4:**
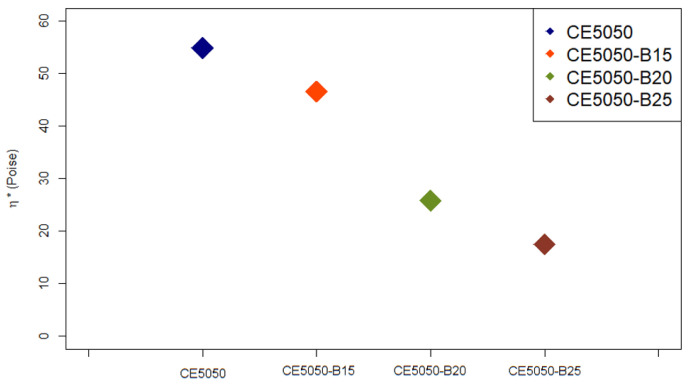
Viscosity $\left(\eta^{*}\right)$ estimation for each investigated blend.

The results obtained for the viscosity $\left(\eta^{*}\right)$ value estimation are reported in Figure 4. Similar results were reported previously by Villanueva et al. [27] in their study on the effect of different concentrations of reactive diluent on epoxy cured by ammine hardener.

These results support the conclusion that all the developed formulations are suitable for the LCD printing process using the LC-Precision Ceramic 3D printer. Hence, each of them was characterized by a $\eta^{*}$ value which is lower than the declared threshold one, i.e., 100 Poise. Furthermore, the additional 15 %wt, 20 %wt, and 25 %wt of oxirane resin resulted in a reduction of about 15%, 53%, and 68%, respectively, in the investigated parameter when compared to the blend composed just by the commercial Cream Hard resin and the epoxy system. Thus, further improvements in terms of resin processability, which is expected to be more fluid, should be expected.

### 3.3. Speed Cure Test

**Figure 5:**
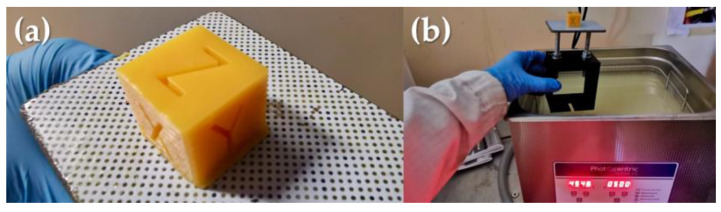
Experimental setup: (**a**) LCD-printed part; (**b**) Washing unit.

We conducted an in-depth investigation of the photocuring process for a single layer, focusing on the CBE5050-B15 blend. The speed cure test results were summarized in Table 4, providing valuable insights into exposure times for optimal curing. These findings will help optimize the printing process, ensuring efficient and accurate production of 3D printed objects using the formulated blends, as the calibration cube (10 × 10 × 10) mm^3^ shows in Figure 5.

The resin with a diluent content of 15 wt% (CE5050-15) was chosen, with an exposure time of 15 s, and a printing profile having the parameters listed in the Table 5.

In addition to the exposure time, the Z lift and Z retract speeds were also increased compared to the base CE5050 formulation, aligning these values with the neat Cream (C) resin. Specifically, the Z lift speed was raised from 10 up to 15 mm/min, and the Z retract speed was increased from 10 up to 50 mm/min.

Therefore, taking the reference sample studied in the previous work [16], i.e., a block of 78 cm^3^, the estimated print time for the CE5050-B15 is approximately 4 h and 20 min, compared to 7 and 16 h for Cream (*C*) and CE5050, respectively.

Once the 3D printing process was accomplished, the post-processing of the manufactured object was performed by using the ultrasonic bath provided by Photocentric for cleaning and removing any residual unpolymerized liquid resin from the surface. Then, the thermal curing phase was carried out following the PC220 cycle. Dimensional measurements were taken for the calibration cube, as reported in Figure 5, before and after exposure to the thermal curing cycle. No significant shrinkage phenomenon was observed. The absence of measurable shrinkage is due to the design choice to geometrically secure the printed part on the print platform even during the thermal curing step or to use 3D printed supports that are integrated with the model when the base cannot be introduced into the oven.

## 4. Discussion

In this study, we investigated novel formulations by introducing epoxy resin and an additional low-viscosity component based on oxirane, mono[(C12-14 alkyl) methyl] derivatives (selected for the role of diluent) to the commercially available LCD Cream resin.

Among the various formulations studied, the CE5050-B15 blend stood out as the most promising, exhibiting superior thermo-mechanical properties compared to the photocured Cream resin. Specifically, the CE5050-B15 blend displayed a significantly higher T_g_ of 115 °C, whereas the photocured Cream resin exhibited a T_g_ of 45 °C. The here-achieved increment for the T_g_ value was also significantly higher than the one achieved by Chen et al. [28], who increased this parameter from 74.8 up to 89.8 °C for a commercial ink for light-curing 3D printing, by using 4-hydroxybenzoate 4-carboxylic phenyl ester as the mesocrystalline unit, so proving the thermal properties of 3D printed products.

The incorporation of the B component into the blend results in faster and more efficient printability by also avoiding volume shrinkage effects. Indeed, the latter phenomenon is generally caused by increasing the quantity of reactive diluent to reduce the viscosity, as found by Deng et al. [29], who used isobornyl methacrylate (IBMA) as a reactive diluent to tailor the resin formulation’s viscosity, which caused a volume shrinkage ranging between about 2 and 6%. Similar results were found by V. Kiliç et al. [30], who investigated the effect of three functional acrylate monomers on volume shrinkage. The addition of the oxirane, mono[(C12-14 alkyl) methyl]-based resin as diluent allowed proper tailoring of the blend’s viscosity and achievement of a reasonable 3D printing process duration without having to resort to alternative solution, i.e., by controlling and increasing the bath temperature to further reduce the resin’s viscosity. By way of example, the latter solution was exploited by Kuhnt et al. [31], who developed a low-cost transparent indium-tin-oxide (ITO)-based heating stage usable with an already-existent DLP machine to control the temperature for each layer that is photocured. However, this solution is not universally usable, since most commercial LCD/DLP 3D printers are not equipped with a thermally controlled bath.

The observed *T*_g_ values may appear distant from those reported by Shen et al. [32] for epoxy-based (DGEBA) resins mixed with various polymeric fillers such as poly(phenylene ether), poly(styrene), poly(methyl methacrylate), and poly(ethylene oxide), where the authors found T_g_ values exceeding 160 °C. However, it is crucial to note that the 3D printing technique employed in this study is different, utilizing Direct-Ink-Writing (DIW). The investigated blends exhibiting excellent thermo-mechanical performance may be well suited for DIW but not necessarily for LCD. Additionally, it should be emphasized that the authors 3D printed a 30 g object in 90 min with DIW, demonstrating a productivity rate of approximately $17\;cm^{3}/h\;$ (assuming an average density of $1.2\;g/cm^{3}$), compared to the approximately $18\;cm^{3}/h$ achieved in the present study. Nevertheless, it is important to consider that this value is likely to increase when populating the print bed with several parts, as the printing time remains constant in the LCD process.

## 5. Conclusions

In conclusion, we have developed epoxy–acrylate blends for the LCD 3D printing technique, taking into account the processability of blends in a dual-curing approach.

The control of the diluent content and of the curing temperature allowed tailoring of the thermomechanical properties of the resin while enhancing its processability. The total build time was reduced by 73% compared to the previous work, so enhancing the LCD printing productivity rate without negatively compromising the properties for the final 3D printed artifact.

Moreover, this strategy opens up new possibilities for dual-curing tailored approaches.

These blends may be of interest for the fabrication of tooling for composites, particularly with out-of-autoclave prepregs that have the capability to cure below 120 °C. Further developments on the use of novel bio-based materials suitable for this 3D printing technology are worthy of investigation. Indeed, the development of environmentally friendly strategies with the aim to reduce the environmental impacts associated with LCD printing is becoming crucial. This is due to the fact that acrylate resins are the main used materials, thus posing challenges in terms of recyclability, because of the non-recyclability associated with their nature.

Additional avenues for exploration may involve refining the LCD process, such as optimizing the mixing method, currently conducted outside the VAT. In fact, while considering extended print times, it may be worthwhile to explore further mixing strategies by developing ad hoc in situ methods, with the aim to achieve well-mixed and homogeneous blends. The latter approach would pave the way for the minimization of material usage, since it would be not required to completely fill the VAT, which is an already valuable approach to reduce waste and environmental impact in the VP (Vat Photopolymerization) 3D printing sector.

## Figures and Tables

**Table 1:** Resin formulations investigated.

ID Resin	Cream Resin wt% Content	Epoxy Resin wt% Content	Oxirane, mono[(C12-14 alkyl) methyl]-Based Resin wt% Content
CE5050	50	50	0
CE5050-B15	50	50	15
CE5050-B20	50	50	20
CE5050-B25	50	50	25

**Table 2:** Viscosity values for each blended component.

ID Resin	$\mathrm{Viscosity}\;(\eta^{*}$) (Poise)
E	130 *
E + 15 %wt B	110
E + 20 %wt B	60
E + 25 %wt B	40
C	1.8 *

* The values with asterisks indicate they originate from Tosto et al. [16].

**Table 3:** *T*_g_ values for post-cured resin blends.

	*T*_g_ [°C]	
ID Resin/ID Cycle	PC180	PC220
CE5050-B15	111.7 ± 0.4	115.4 ± 0.9
CE5050-B20	101.1 ± 0.5	107.5 ± 0.7
CE5050-B25	90.8 ± 0.4	99.4 ± 1.0

**Table 4:** Average mean values of layer thickness and width overcure from the printing trials on a square prism for LCD printing.

Exposure Time (s)	Thickness (μm)			Width Overcure (*%*)		
	CE5050-B15	CE5050-B20	CE5050-B25	CE5050-B15	CE5050-B20	CE5050-B25
5	179	221	209	8.60%	8.20%	8.10%
10	251	247	277	7.50%	7.20%	7.20%
15	286	281	318	8.10%	8.40%	7.00%
20	324	332	350	8.00%	9.70%	8.10%
25	377	367	410	7.70%	6.80%	7.10%
30	406	409	450	6.90%	5.20%	8.60%

**Table 5:** LCD 3D printing process setting for the CE5050-B15 formulation.

ID Resin	Exposure Time (s)	Z Lift Distance (mm)	Z Lift Speed (mm/min)	Z Retract Speed (mm/min)
CE5050-15	15	3	15	50

## Data Availability

Data are contained within the article.
